# Transcriptomic analysis of reproductive damage in the epididymis of male Kunming mice induced by chronic infection of *Toxoplasma gondii* PRU strain

**DOI:** 10.1186/s13071-019-3783-2

**Published:** 2019-11-08

**Authors:** Yu-Xiang Zheng, Xiu-Xiang Zhang, Jorge A. Hernandez, Yasser S. Mahmmod, Wan-Yi Huang, Gui-Feng Li, Ya-Pei Wang, Xue Zhou, Xiu-Ming Li, Zi-Guo Yuan

**Affiliations:** 1 College of Veterinary Medicine, South China Agricultural University, Guangzhou, 510642 Guangdong People’s Republic of China; 2 Key Laboratory of Zoonosis Prevention and Control of Guangdong Province, Guangzhou, 510642 People’s Republic of China; 3 College of plant, South China Agricultural University, Guangzhou, 510642 Guangdong People’s Republic of China; 4 College of Veterinary Medicine, University of Florida, 2015 SW 16th Avenue, Gainesville, FL 32610-0136 USA; 5 Infectious Diseases, Department of Animal Medicine, Faculty of Veterinary Medicine, Zagazig University, Zagazig, Sharkia Province 44511 Egypt; 6 IRTA, Centre for Research into Animal Health (CReSA-IRTA), Campus of Autonomous University of Barcelona, Bellaterra, 08193 Barcelona, Spain; 7 College of Animal Science and Technology, Heilongjiang BaYi Agricultural University, Daqing, 163319 People’s Republic of China

**Keywords:** *Toxoplasma gondii*, Chronic infection, Reproductive impairment, DEGs, RNA-seq

## Abstract

**Background:**

Some researchers have reported that *Toxoplasma gondii* can cause serious reproductive impairment in male animals. Specifically, *T. gondii* destroy the quality of sperm in the epididymis, which affects their sexual ability. However, among such studies, none have investigated the male reproductive transcriptome. Therefore, to investigate the relationship between *T. gondii* and sperm maturation, we infected mice with *T. gondii* prugniaud (PRU) strain and performed transcriptome sequencing of the epididymis.

**Results:**

Compared with the control group, 431 upregulated and 229 downregulated differentially expressed genes (DEGs) were found (*P*-value < 0.05, false discovery rate (FDR) < 0.05 and |log2 (fold change)| ≥ 1). According to results of a bioinformatics analysis, Gene Ontology (GO) function is divided into three categories: cellular component, molecular function and biological process. Upon performing GO analysis, we found that some DEGs correlated with an integral part of membrane, protein complex, cell surface, ATP binding, immune system process, signal transduction and metabolic process which are responsible for the epididymal injury. DEGs were mapped to 101 unique KEGG pathways. Pathways such as cytokine-cytokine receptor interaction, glycolysis/gluconeogenesis and apoptosis are closely related to sperm quality. Moreover, *Tnfsf10* and *spata18* can damage the mitochondria in sperm, which decreases sperm motility and morphology.

**Conclusions:**

We sequenced the reproductive system of male mice chronically infected with *T. gondii*, which provides a new direction for research into male sterility caused by *Toxoplasma* infection. This work provides valuable information and a comprehensive database for future studies of the interaction between *T. gondii* infection and the male reproductive system.

## Background

*Toxoplasma gondii* is an intracellular parasite that infects almost all warm-blooded animals [1, 2]. Pregnant women infected with *T. gondii* can transmit the infection to their fetus through vertical transmission resulting in abortion, fetal abnormalities and death [3]. Additionally, *Toxoplasma* infection can cause damage to the male reproductive system, such as sexual dysfunction and infertility. The epididymis is an important accessory organ of the male reproductive system, where sperm maturation and storage take place. Sperm maturation in the epididymis is a highly programmed process which may be affected by the epididymis microenvironment. Therefore, studying the characteristics of the epididymis is important for identification of factors affecting sperm maturation. Previous studies have shown that reproductive pipeline damage [4] and hypogonadism [5] are associated with *T. gondii* infection. Furthermore, a large number of tachyzoites in semen of infertile patients and anti-sperm antibodies (AsAb) were detected in *T. gondii* infection [6]. Other studies have reported more advanced pathological changes in *T. gondii* infection cases, such as granular degeneration of vas deferens epithelial cells, luminal stenosis and interstitium infiltration with inflammatory cells [7]. The density and motility rate of sperm in epididymis infected with *T. gondii* were significantly lower than the control group; however, the rate of sperm deformity was increased [8, 9].

Recently, various -omics technologies, including transcriptomics, proteomics and metabolomics, have been developed [10]. Transcriptomic analysis is one of the most common high-throughput techniques, which identify the types and copy numbers of mRNAs, while the cells are living in a functional state [11]. Genetic studies have shown that mRNA act as a “bridge” for genetic information transmission between DNA and protein. Hence, it is a valuable source to identify the expression of all genes with a specific time and space in the cell.

Previous studies have investigated the association between *T. gondii* and male infertility using epidemiology, pathology and serology methods and techniques [12], detection of *Toxoplasma* DNA in semen [13] and observation of the tissue and cell damage [14]. To our knowledge, studies investigating the differentially expressed genes of host and *T. gondii* by transcriptome sequencing of the male reproductive system are limited. The main objective of this study was to examine differential gene expression by RNA sequencing (RNA-seq) technology in order to identify key genes associated with *T. gondii* (PRU strain) chronic infection of the epididymis in male Kunming mice.

## Methods

### Study population and experiment set-up

Thirty specific-pathogen-free eight-week-old Kunming male mice were purchased from the Laboratory Animal Center of Guangdong Province. Fifteen mice were subjected as the experiment groups, and 15 mice were subjected as the control group (to reduce individual differences, we set the epididymal tissue of 5 mice as a biological replicate, the experiment group and the control group were repeated three times). Mice in the experimental group were inoculated with four cysts of *T. gondii* PRU strain diluted with normal saline to 0.5 ml through the intragastric administration route (we have previously studied the optimal number of PRU strains infections per mice, which tried to minimize the case damage caused by *T. gondii* infection in mice). Meanwhile, the control groups were given the same amount of normal saline only. The male mice were sacrificed at 35 days post-infection. Under sterile conditions, the epididymides were harvested. Under the microscope, the peripheral adipose tissue and blood vessels of the harvested epididymides were carefully removed. The processed epididymides were subjected to quick freezing by storing them in liquid nitrogen at – 80 °C for subsequent analysis.

### Transcriptome sequencing, data analysis and verification

At present, transcriptome sequencing is a mature high-throughput second-generation sequencing method. A brief description of the experimental process is provided in Table 1; detailed methods of transcriptome sequencing, data analysis and verification can be found in Additional file 1: Text S1.

**Table 1:** Procedures for transcriptome sequencing, data analysis and validation

Experimental procedure	Experimental method or threshold considered
Extraction of total RNA	Trizol reagent (Invitrogen, CA, USA)
Quality and purity detection of total RNA	RNA 6000 Nano LabChip Kit (Agilent, CA, USA) with RIN number > 7.0
cDNA library construction	The cleaved mRNA fragments were reverse-transcribed to create the final cDNA library by the instructions of the mRNA-Seq sample preparation kit (Illumina, San Diego, USA)
Sample library construction	Illumina Hiseq 2500 sequencing platform
Transcript abundance estimation	The RPKM of each gene were calculated by the MARS model in DEGseq program, when the RPKM greater than 1000, it is considered to be a highly expressed gene
Threshold of differentially expressed genes	FDR (false discovery rate) < 0.001 and |log2fold change| ≥ 1
Bioinformatics analysis	GO and KEGG enrichment
Verification of mRNA expression level	Real-time fluorescence quantitative PCR
Verification of protein expression level	Western blot

## Results

### Pre-experiment

Before the histological analysis, we conducted a preliminary study on the sperm quality of *T. gondii* infection, including the statistics of sperm survival rate and total sperm count. The sperm survival rate was calculated according to the formula: Sperm survival rate = (Total sperm count - Dead sperm count)/Total sperm count × 100%; three experiments were carried out in each group. The results showed that the sperm survival rate of the experimental group (32%) was significantly lower than that of the control group (74.67%).

The sperm count per milliliter semen was calculated according to the formula: Sperm count per milliliter semen = (5 medium square count/80) × 400 × dilution multiple × 10,000. We diluted sperm 10 times for microscopic examination. The experimental data are shown in Additional file 2: Figure S1; there were significant differences in total sperm count between the experimental group and the control group (*P* = 0.0058). The data showed that *T. gondii* infection had a negative effect on sperm quality.

### Identification of infection in mice

The outward appearance changes of infected mice comprised mainly a stress reaction about a week after infection, which included depression and the fur is not smooth. Thirty days after infection, 20 mice were selected for tail vein blood collection and qPCR was used to verify the infection of mice (*T. gondii* ROP18 virulence gene was detected and using the β-actin as a reference); the result is shown in Additional file 3: Figure S2. All of the 20 mice were infected. Fifteen mice were randomly selected for dissection and epididymis tissue was quickly removed. Meanwhile, the mouse brain was grinded and diluted with 100 µl saline solution; 5 µl of brain homogenate was taken for microscopic examination and the number of cysts was counted. Cysts from each mouse were counted three times and averaged. Statistical results showed that 5 µl brain homogenate contained three or more cysts. Therefore, we determined that all of the experimental mice were infected with *T. gondii.*

### Quality analysis of RNA

The purity of total RNA was high, and the ratios OD_260_/OD_280_ and OD_260_/OD_230_ were both higher than 2.0 (Table 2). The RNA integrity number (RIN) was higher than 7, and the ratio of *28S*/*18S* rRNA was higher than 0.7. This indicated that the RNA had good integrity and met the requirements of the subsequent experiments.

**Table 2:** Quality analysis of RNA

Sample	Concentration (µg/µl)	O.D. 260/280	O.D. 260/230	Amount (µg)	rRNA	RIN	QC evaluation
					*28S*/*18S*		
PRU-1	1.46	2.07	2.3	65.83	0.8	7	A
PRU-2	1.16	2.09	2.25	52.24	1.8	7.2	A
PRU-3	1.38	2.08	2.28	62.34	1.5	7.5	A
Control-1	0.87	2.07	2.35	39.35	0.8	7	A
Control-2	1.69	2.1	2.32	76.25	1.7	7.9	A
Control-3	1.26	2.03	2.29	56.65	1.8	8.1	A

### Sequencing data preprocessing

The sample library constructed by Illumina Hiseq 2500 sequencing platform. We obtained 321,183,388 raw reads (48.17G). After trimming, we obtained 314,632,244 clean reads (47.21G). Among them, the amounts of sequencing data of each sample were more than 6G. In high throughput sequencing, each measurement of a basic group gives a corresponding value, which is a measure of the accuracy (Table 3).

**Table 3:** Pre-treatment results of data quality in the high throughput sequencing

Sample	Raw data reads	Base	Valid data reads	Base	Valid reads %	Q20%	Q30%	GC%
PRU-1	42,640,370	6.4G	41,484,632	6.22G	97.29	98.77	90.81	46.5
PRU-2	49,368,291	7.4G	48,326,910	7.25G	97.89	98.47	90.93	48.0
PRU-3	43,890,326	6.58G	42,897,451	6.44G	97.74	98.93	90.25	47.7
Control-1	62,208,188	9.33G	60,729,140	9.11G	97.62	98.86	91.00	47.5
Control-2	58,037,289	8.7G	57,291,719	8.6G	98.71	98.72	90.35	50.0
Control-3	65,038,924	9.76G	63,902,392	9.59G	98.25	99.04	91.37	46.5

*Notes*: Q20%, error rate is 1%, or the correct rate is 99%; Q30%, error rate is 0.1%, or the correct rate is 99.9%

### Analysis of gene expression

**Fig. 1:**
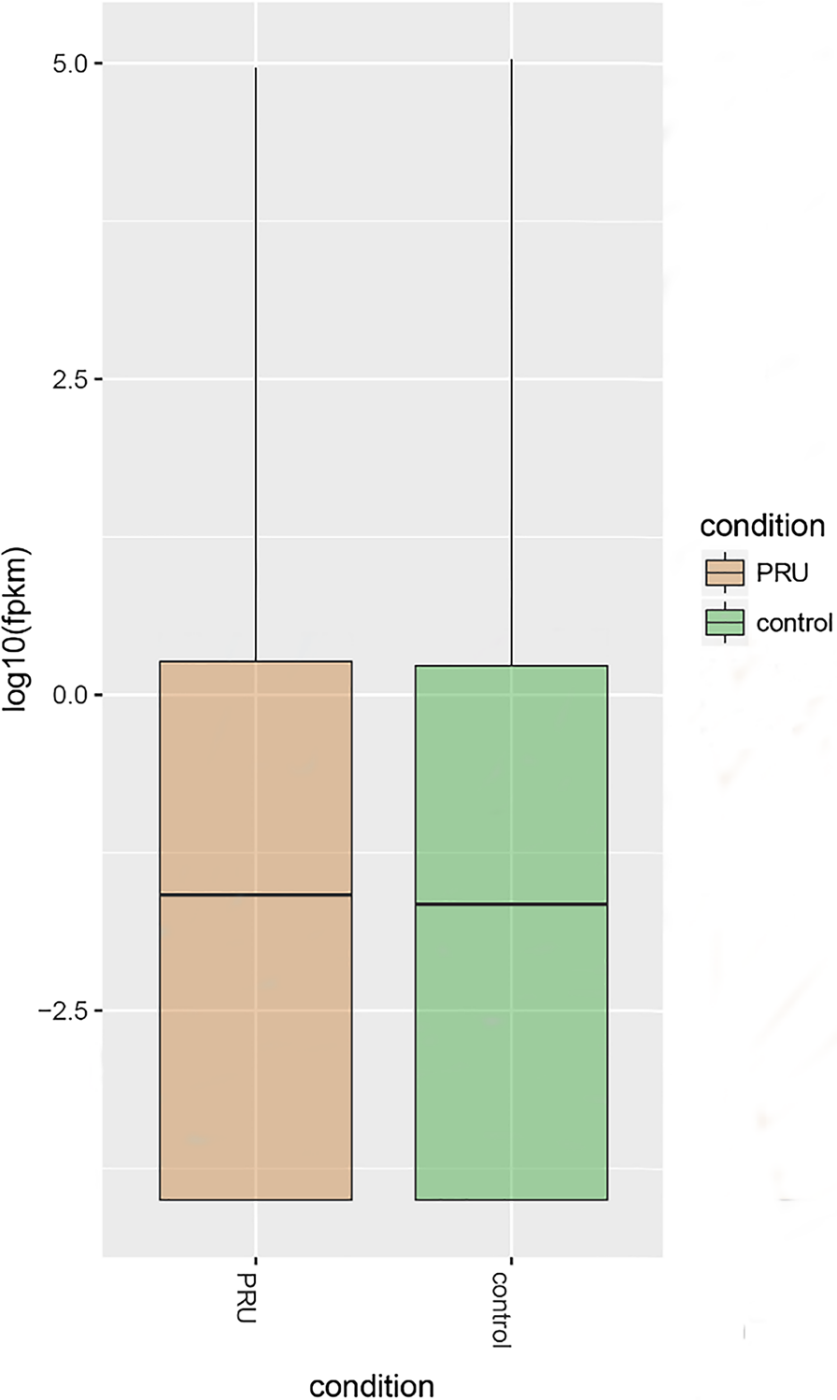
Statistics of gene expression distribution. Infection group: the maximum, upper quartile, median, lower quartile and the minimum values are 92252.6, 1.84, 0.03, 0 and 0, respectively. Control group: the maximum, upper quartile, median, lower quartile and minimum values are 107446, 1.7, 0.02, 0 and 0, respectively

RPKM/FPKM was used to measure the abundance of gene expression in the transcriptome. We used FPKM to estimate the abundance of known genes expresses in different samples. After the construction of new transcripts, 69,907 transcripts and 29,128 unigenes were obtained. The five-demographic data (distribution) of experimental and control groups are shown in Fig. 1.

### Analysis of DEGs

**Fig. 2:**
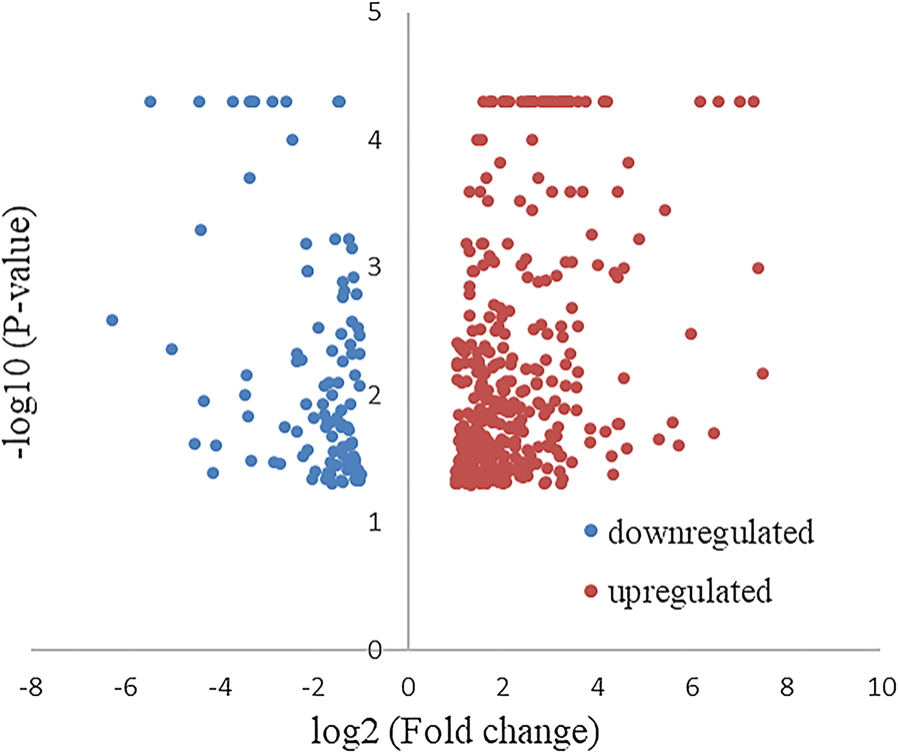
Distribution map of significant differentially expressed genes. Bright red dots indicate significantly upregulated genes, while blue dots indicate significantly downregulated genes

Significant DEGs from the differential genes with *P* < 0.05, |log2 (fold change)| ≥ 1 and the FDR corrected *P-*value < 0.05 were selected. The overall distribution of DEGs was observed by drawing a volcano plot (Fig. 2). Compared with the control group, the number of up- and downregulated genes was 431 and 229, respectively. Partial DEGs are listed in Tables 4 and 5.

**Table 4:** Partial of upregulated DEGs in the high throughput sequencing data

Symbol	Name	log2(FC)	Function
Iigp1	Interferon-inducible GTPase 1	6.5608	Required for disruption of the parasitophorous vacuole formed following *T. gondii* infection and subsequent killing of the parasite
Klrb1c	Killer cell lectin-like receptor subfamily B member 1C	6.17868	Plays a stimulatory role on natural killer (NK) cells cytotoxicity, activation by cross-linking of the receptor induces Ca^2+^ mobilization and interferon-gamma production
Tgtp1	T cell-specific GTPase 1	5.61362	Involved in innate cell-autonomous resistance to intracellular pathogens, such as *T. gondii*
Tnfsf10	Tumor necrosis factor (ligand) superfamily, member 10	1.53097	Involved in male gonad development and regulation of the apoptotic process
Spata18	Spermatogenesis associated 18	1.29567	A key regulator of mitochondrial quality that mediates the repairing or degradation of unhealthy mitochondria in response to mitochondrial damage
Pgk2	Phosphoglycerate kinase 2	1.11099	Essential for sperm motility and male fertility but is not required for the completion of spermatogenesis
Piwil2	Piwi-like RNA-mediated gene silencing 2	1.1907	Essential role in meiotic differentiation of spermatocytes and self-renewal of spermatogonial stem cells

**Table 5:** Partial of downregulated DEGs in the high throughput sequencing data

Symbol	Name	log2(FC)	Function
Lipf	Gastric triacylglycerol lipase	− 3.40022	Involved in lipid catabolic process and mitochondria when classification of cellular component
Nrk	Nik-related protein kinase	− 3.41519	Involved in the TNF-alpha-induced signaling pathway
Nwd1	NACHT and WD repeat domain containing 1	− 1.60721	Play a role in the control of androgen receptor (AR) protein steady-state levels
Slc14a2	Solute carrier family 14 (urea transporter), member 2	− 1.11457	Mediates rapid transepithelial urea transport across the inner medullary collecting duct and plays a major role in the urinary concentrating mechanism
Spink2	Serine protease inhibitor Kazal type 2	− 1.02159	Required for maintenance of normal spermatogenesis, involved in regulating serine protease-dependent germ cell apoptosis

### Bioinformatics analysis for the DEGs

**Fig. 3:**
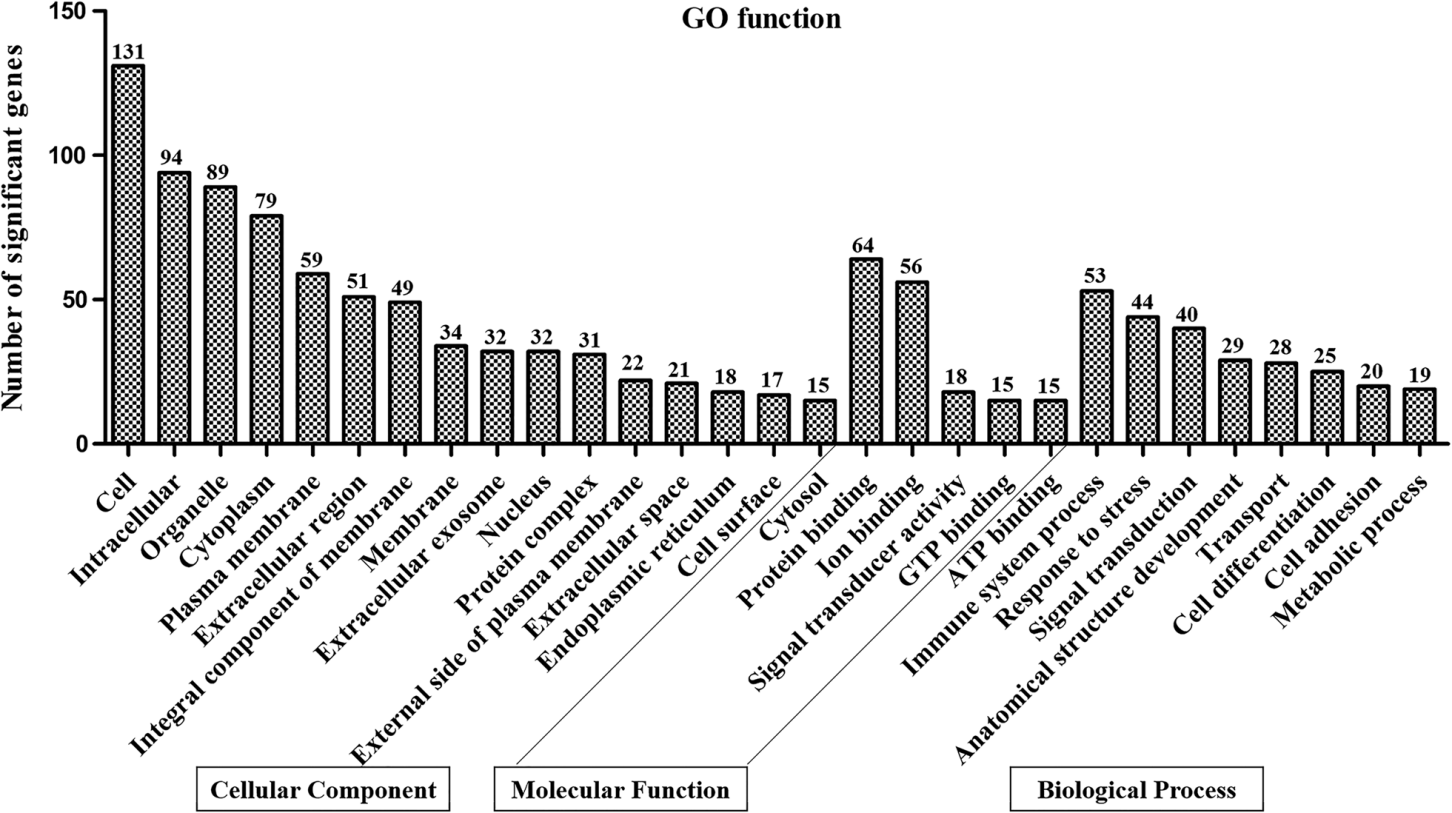
GO analysis of the significant DEGs

GO database analysis was conducted on the DEGs, including biological processes (1746), cellular components (1275) and molecular functions (785); the distribution diagram of GO terms with significant changes in classification is shown in Fig. 3. The main categories in the biological processes include immune system process, response to stress and signal transduction. As for the cellular components, the GO terms with the most differentially expressed genes were cell, intracellular and organelle. The molecular functions were involved mainly in the protein binding followed by ion binding, and transmembrane transporter activity.

**Fig. 4:**
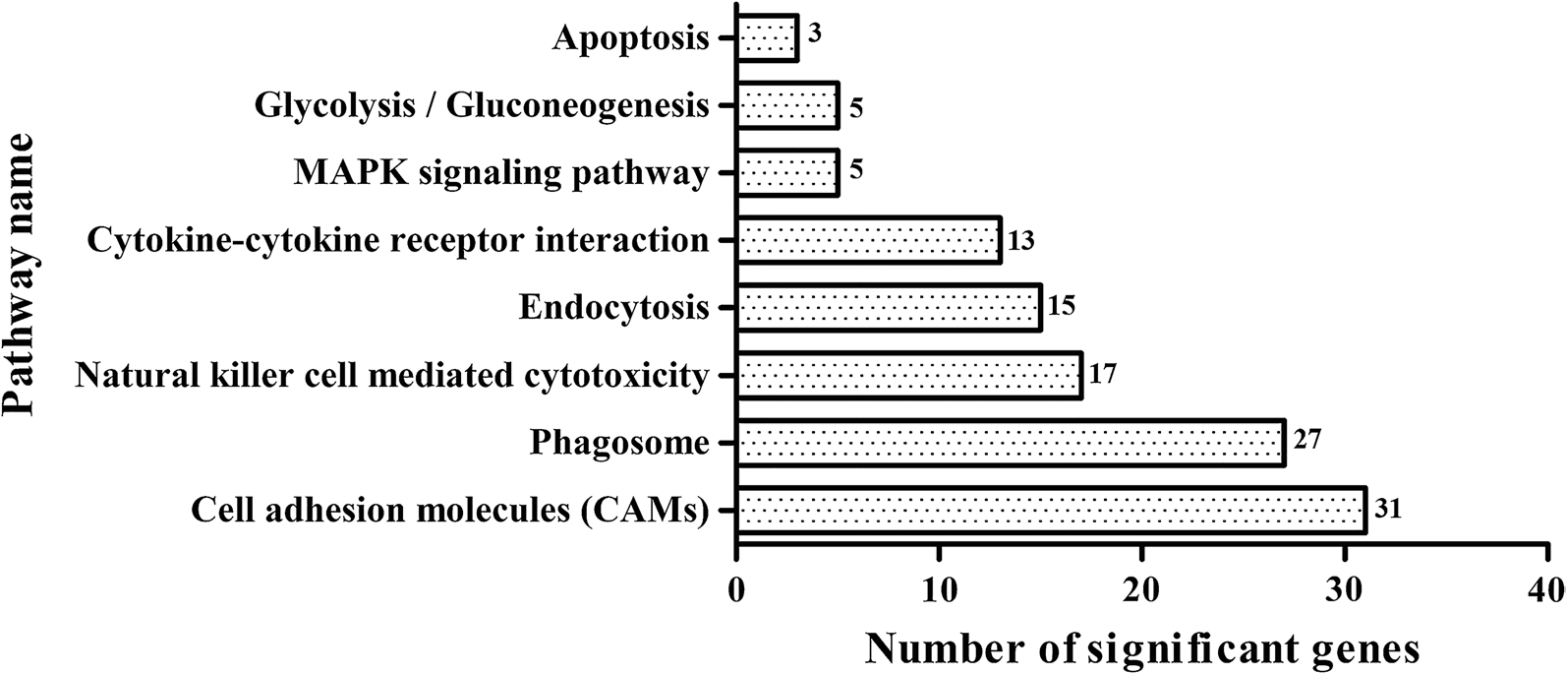
The most enriched pathways of DEGs

KEGG database analysis showed that the DEGs were enriched in 101 pathways. The most enriched pathways of DEGs are exhibited in Fig. 4. Markedly, cell adhesion (31), phagosome (27) and natural killer cell mediated cytotoxicity (17) were the major three enriched pathways (Fig. 4). However, the pathway directly related to male reproduction was not found.

### Verification results by qPCR analyzing and western blotting

**Fig. 5:**
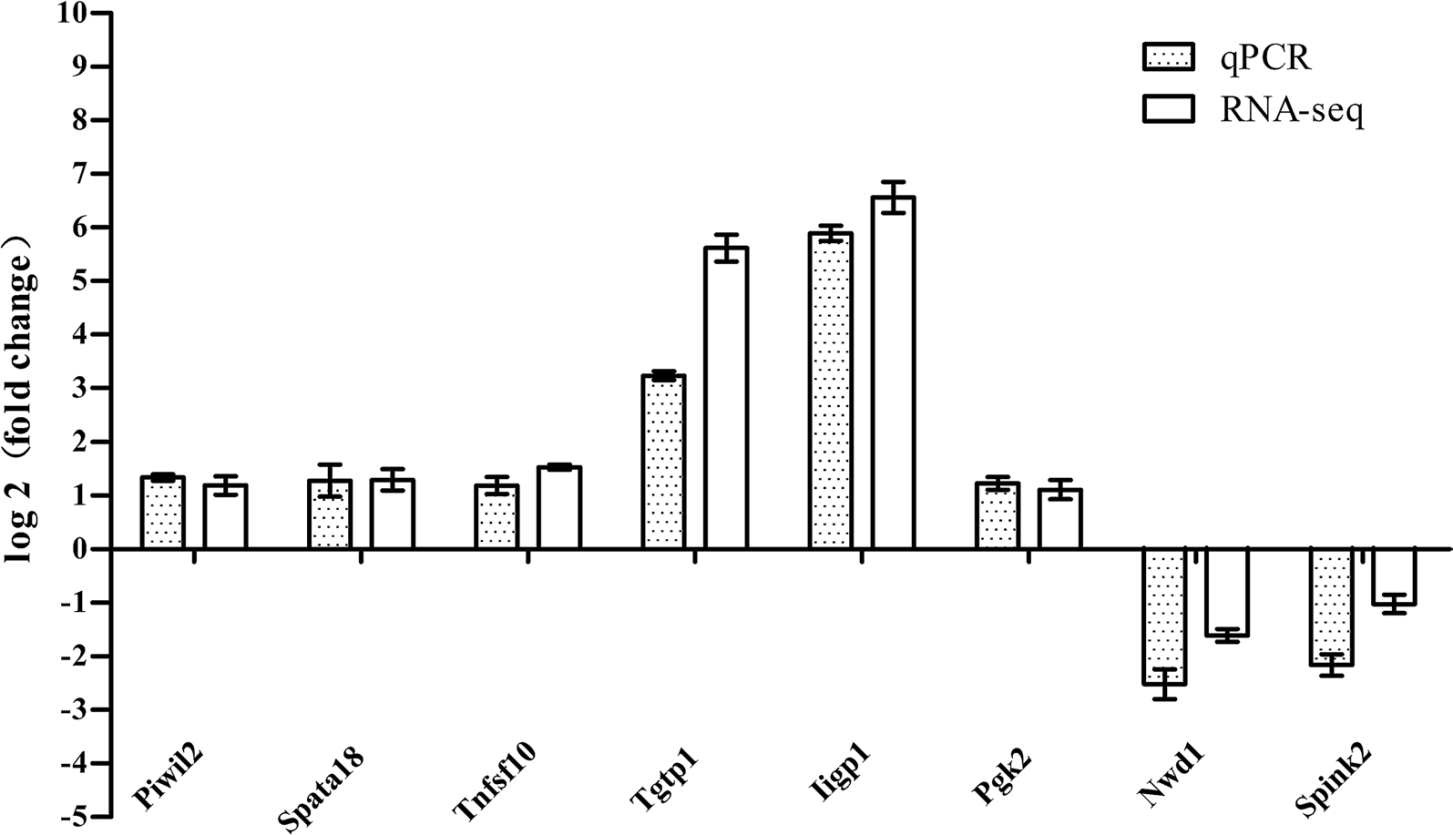
Results of qPCR and compared with RNA-seq

**Fig. 6:**
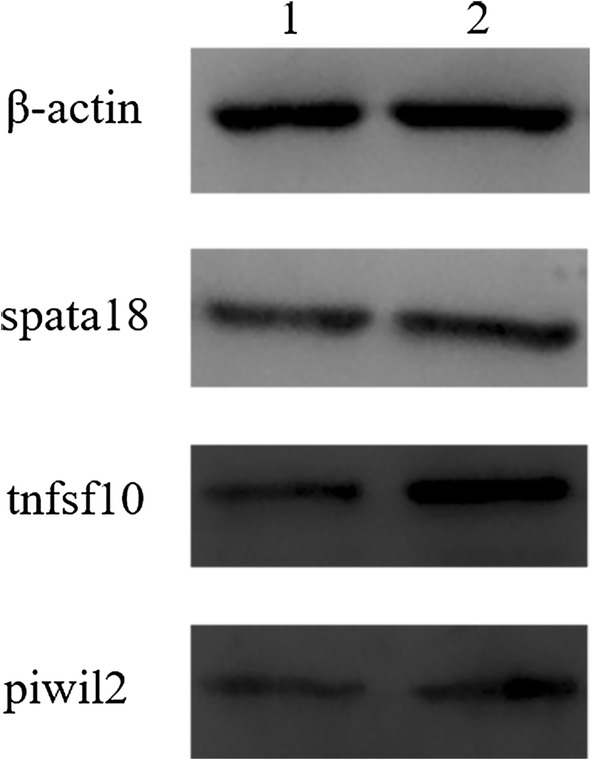
Results of Western blot compared with RNA-seq. Lane 1: control group; Lane 2: infection group; from top to bottom, represent the *β*-actin internal reference, spata18, tnfsf10 and piwil2 protein

The eight genes related to reproduction were identified by qPCR (Table 6); these expression trends were coincident with RNA-seq results (Fig. 5). The result of qPCR did not coincide with the RNA-seq perfectly; however, it is still possible to increase the reliability of these genes to a certain extent. DEGs including *Piwil2*, *Spata18* and *Tnfsf10* were selected for Western blot analysis. The results of the Western blot showed the same changing trend as the results of RNA-seq, although the specific amount of expression was different (Fig. 6).

**Table 6:** Design of primers used for qPCR analysis

Gene ID	Gene	Forward primer	Reverse primer	Product length (bp)
57746	piwil2	GGCAGAGGCCTTGTGTTTAG	CGTTTTGAAGGAGGCCGGAA	134
73472	Spata18	TTATCACGTGTGGCCTGCTC	TTAAGCTCCTGCTACGGCAC	125
22035	Tnfsf10	ACGCTTCCAAGATGGTCTCA	ACAGTCCGTACTCGGCATCT	147
21822	Tgtp1	CGCCACGTCTTCTCACTGTC	CACCAAGTGGAATGGTGGCT	130
60440	Iigp1	TCACTATGACTTCCCCGTCCT	TGCTTCAGAAATTGCCGCTT	132
18663	Pgk2	GCCTGTGCCAACCCAGATAA	ACAGTGATGCTTGGAAGGCT	145
319555	Nwd1	CTCTTTGGACCACCAGGCAT	AGGCTCAGCTTTGATGTCCC	125
69982	Spink2	GCATGCCCTAGGAACCTCAA	ATATGGCTACCGTCCTCCCT	101
11461	β-actin	AGAGAAGCTGTGCTATGTTGCT	GGAACCGCTCGTTGCCAATA	128

## Discussion

We investigated the pathogenesis of *T. gondii* in the male reproductive tract of mice at the stage of sperm maturation using RNA-Seq technology. Additionally, we performed enrichment methodologies analysis to screen the DEGs that might be related to the reproductive functions. Results were confirmed by using qPCR and Western blot methods. Our findings showed that the RNA-Seq is accurate.

The analysis of DEGs suggested that some genes were interrelated with the infection of *T. gondii*, such as *Iigp1* (interferon-inducible GTPase 1), *Tgtp1* (T-cell-specific guanine nucleotide triphosphate-binding protein 1) and *Tgtp2. Iigp1* found in the parasitophorous vacuoles (PV) plays a role in the destruction of PV and death of the parasite, when the *Iigp1* gene is overexpressed [15]. We found that *Iigp1* was in regulation of autophagy in the GO classification. Hence, it is possible that *Iigp1* is involved in autophagy, although the specific mechanism is not clear. During infection of the type II of *T. gondii*, *Tgtp1* and *Tgtp2* were recruited to the PV membrane, PV vesiculation, rupture and digestion of the parasite within the cytosol [15, 16]. In our study, the *Iigp1*, *Tgtp1* and *Tgtp2* were significantly upregulated by 94-fold, 48-fold and 23-fold, respectively. This could be the result of host resistance to *T. gondii*. However, after *T. gondii* infection, *Tgtp1* and *Tgtp2* had low levels of gene expression abundance. The average abundance of *Tgtp1* was 13.7 for the experimental group and 0.58 for the control group and the average abundances of *Tgtp 2* were 15.39 and 0.31, respectively. *Tgtp1* and *Tgtp2* are mainly expressed in the thymus and lymph nodes, predominantly T-cells and IFNG-stimulated macrophages [17]. In other words, these genes are tissue-specific. After *T. gondii* infection, the immune system was stimulated, and the expression level of these genes was greatly increased.

### Glycolytic pathway and sperm motility

There are several enriched pathways in our data analysis. Glycolysis was thought to be the oldest and most primitive way of biological energy harvesting. We focused on DEGs that affect glycolysis such as phosphoglycerate kinase 2 (*Pgk2*), phosphoglycerate mutase (*Pgam*) and lactate dehydrogenase C (*LDHC*). Phosphoglycerate kinase is the first regulation factor of ATP generated in the glycolysis pathway. In the reproductive system, the examination of sperm in elderly and young male patients with asthenospermia showed that the level of *Pgk2* was reduced. *Pgk2*, which is related to the quality of sperm, is mainly expressed in able-bodied spermatids [18]. *Pgk2* deficiency leads to a decrease in sperm motility and ATP levels [19]. *Pgam* is an important enzyme of glycolysis and gluconeogenesis pathway, catalyzes the 3-phosphoglycerate (3-PGA) into 2-phosphoglycerate (2-PGA), which dominates the balance between glycolysis and gluconeogenesis. Moreover, *Pgam2* is related to the fibrous sheath of the main segment in the sperm flagellum [20]. The fibrous sheath on the sperm flagellum is the source of ATP and an essential structure of sperm motility [21]. Therefore, *Pgam2* is the key determinant to maintaining sperm motility and morphology. *LDHC* is an enzyme that controls the conversion of pyruvate to L-lactic acid, which plays a key role in the process of spermatogenesis and capacitation by participating in cellular energy metabolism [22]. *LDHC* was found in the human spermatozoa and spermatogenic cells and was increased gradually with meiosis. *LDHC* in mature sperm is mainly located in the fibrous sheath of the main segment of the tail of sperm, which is involved in the regulation of sperm motility. In addition, *LDHC* is also participates in the homeostasis of ATP. Deficiency/destruction of germline-specific *LDHC* leads to infertility, including a reduction in sperm ATP content and reduced sperm motility [22, 23]. Conclusively, the enzymes are closely linked with sperm activity and morphology.

In the present study, the DEGs of glycolysis indicated that *T. gondii* infection might also result in dysregulation of those enzymes in humans, thus promoting a negative influence on the quality of male sperm. Normally, the energy production efficiency of oxidative phosphorylation is much higher than that of glycolysis, but glycolytic pathways have their particular importance in sperm energy sources. Mukai & Okuno [24] noticed that when the oxidative phosphorylation was blocked by rotenone of mitochondrial respiratory chain inhibitors, the sperm keeps moving. Therefore, glycolysis is not only an important source of energy for sperm but also plays a role in sperm capacitation [25] and acrosome reaction [26, 27].

### Mitochondrial damage and apoptosis

Spermatogenesis associated 18 (*Spata18*) is a key regulator of mitochondrial quality that mediates repair or degradation of unhealthy mitochondria [28]. There is no pathway for *Spata18*, but previous research has shown that it is a monitor of cell differentiation process, which is involved in the maturation of spermatids into spermatozoa [29].

In our study, the expression of *Tnfsf10* gene was upregulated to 2.89-fold in the pathway of cytokine-cytokine receptor interaction, apoptosis and natural killer cell mediated cytotoxicity. *Tnfsf10* (*Trail*) is a member of the tumor necrosis factor (TNF) superfamily, and is an important signal molecule to maintain the stability of germ cells and the functional spermatogenesis in testis [30]. Apoptosis, or programmed cell death (PCD), is a biological process controlled by genes and plays a vital role in maintaining the morphology of normal tissues and organs. So far, the mitochondrial pathway is considered to be the classical pathway of apoptotic signal pathways. Mitochondria control the life and death of cells in aerobic environments, which is the main place of ATP production, and the major regulatory site of cell apoptosis. Mitochondrial permeability transition pores (MPTP) exist in the inner and outer membranes of mitochondria, and the permeability transition plays a vital role in cell apoptosis [31]. The pathway of cytokine-cytokine receptor interaction shows *Tnfsf10* as a cytokine and interacts with the cytokine receptor *Tnfsf10b*. In the apoptosis pathway, *Tnfsf10b* (2.3-fold upregulation) as a death receptor, interacts with *Tnfsf10* causing production of the death inducing signaling complex (DISC), which initiates the caspaseprotease family (aspartate-specific cysteine proteases) and activates apoptosis through DNA fragmentation, low synthesis of ploy, and destruction of nuclear membrane integrity [32]. At the same time, the translocation and permeabilization of mitochondrial membrane could induce apoptosis through Bax/Bak [33]. MPTP opening resulting from exogenous injury factors (such as Ca^2+^ overload) would lead to necrosis and apoptosis of cells. Some scholars have found that the synergism of melittin with *Trail* could increase the number of apoptosis in hepatoma cell through activating Ca^2+^/calmodulin-dependent protein kinase [34]. This is consistent with the finding of Chen et al. [35] who stated that the Ca^2+^ dependent calpain and/or Ca^2+^ independent cathepsins participate in apoptosis-like PCD and necrosis-like PCD. Trail gene-deficient (Trail2/2) mice were significantly reduced by 54% compared to the wild type mice, which explains the negative effects on sperm maturation in the reproductive system [30]. A high concentration of soluble *Tnfsf10* was detected in the seminal plasma, which revealed a variable expression of *Trail* receptors in the sperm cellular fraction among different participants [36]. Those results suggest that *Tnfsf10* may play an important role in sperm maturation.

The cytotoxicity of natural killer (NK) cells is motivated by the activating cell receptors. It can switch on the cascade reaction of downstream signal transduction. In our study, the Src family kinase (*Fcgr4*) was related to the cytotoxic pathway which is mediated by NK cells. The *Fcgr4* also could active the mitogen activated protein kinase (MAPK) pathway, which could produce the cytotoxicity and secret proinflammatory cytokines (such as IFN-γ, TNF-α and GMCS). The upregulation of *piwi* and *Itgb2* genes mediates the target cell apoptosis by *Tnfsf10*, which is caused by perforin and granzyme. The *Gzmb* (*Granzyme B*) gene is responsible for aspartate-specific cysteine proteases (DNA fragmentation, low synthesis of ploy and destruction of nuclear membrane integrity) which cause apoptosis.

From these pathways, we consider that *Tnfsf10* is directly or indirectly connected with the male reproductive system. Chronic infection of *T. gondii* gives rise to dysregulation genes in these pathways, which in turn have a negative consequence on the integrity and motility of sperm. At the stage where spermatids mature into spermatozoa, mitochondria formed the mitochondrial sheath by gathering around the tail and wrapping around the shaft spirally. Finally, it can provide the energy for rotational motion of the tail in the way of making full use of energy in plasma drops. The fertility was influenced by various channels to create the apoptosis of germ cells.

### Testis-specific genes in the epididymis

Our data analysis revealed that many DEGs were upregulated in the epididymis; however, previous studies have shown that they are testicular-specific genes, such as *Piwil2* and *RGS22* (regulator of G-protein signaling 22) genes. *Piwil2* is a member of the Argonaute family and PIWI (the P-element-induced wimpy testis) subfamily. PIWI family proteins are only expressed in animal gonad tissues and have tissue specificity, these are closely related to the maintenance of germ stem cells and spermatogenesis [37]. The *Piwil2* gene, expressed in tumor stem cells and germ cells, plays an important role in the self-renewal of stem cells, embryo formation and transcriptional regulation [38]. Similarly, *Rgs22* is a specific gene in the male testis, which can be expressed simultaneously in a variety of epithelial tumors. In testis, *Rgs22* protein is associated with spermatogenesis, which can be expressed in spermatogenic cells involved in the spermatogonia differentiation into sperm cells and Sertoli cells. Hu et al. [39] found that the expression of *Rgs22* was downregulated in patients with azoospermia. Proteins are located in spermatogenic cells and Leydig cells, which interact with the guanine-nucleotide-binding proteins [39]. Thus, we consider that *Rgs22* may be related to the meiotic stage of spermatogenesis.

In summary, *Piwil2* and *Rgs22* genes are testis-specific genes, mainly expressed in spermatogenic cells and play a key role in the spermatogenesis process. Other genes that were identified (see Table 7) are testis-specific and highly expressed in the epididymis. One possible reason for the latter observation is the invasion of *T. gondii*, which contributes to the destruction of the blood-testis barrier of the testis, and disruption of the spermatogenesis homeostasis [40]. Furthermore, spermatogenic cells in the seminiferous tubules were disordered, resulting in the increase of testis-specific gene expression in the epididymis. However, the epididymis has its tissue-specific microenvironment [41, 42] which is different from that in the testis. The microenvironment in the epididymis is not appropriate to regulate the cells in the testis and therefore, the specific mechanism is unclear and needs further investigation.

**Table 7:** The testis-specific gene was highly expressed in the epididymis

Symbol	log2 (FC)	Tissue specificity	Function
Piwil2	1.1907	Expressed in adult testis, specifically in spermatocytes and in spermatogonia	Plays an essential role in meiotic differentiation of spermatocytes and self-renewal of spermatogonial stem cells
Rgs22	2.18187	Expressed in testis, including in Leydig cells and spermatogenic cells from the spermatogonia to spermatid stages	Negative regulation of signal transduction
Shcbp1l	1.78449	Testis-specific. Expressed in pachytene spermatocytes and elongating spermatids inside the seminiferous tubules	Testis-specific spindle-associated factor that plays a role in spermatogenesis
Spz1	1.08852	Expressed specifically in the testis (Sertoli and Leydig cells) and epididymis	Play an important role in the regulation of cell proliferation and differentiation during spermatogenesis
Spata18	1.29567	In testis, expressed primarily in spermatids	Key regulator of mitochondrial quality that mediates the repairing or degradation of unhealthy mitochondria in response to mitochondrial damage

## Conclusions

We sequenced the reproductive system of male mice chronically infected with *T. gondii*, which provides a new direction for research into male sterility caused by *Toxoplasma* infection. This work provides valuable information and a comprehensive database for future studies of the interaction between *T. gondii* infection and the male reproductive system.

## Supplementary information

**Additional file 1: Text S1.** Detailed methods of transcriptome sequencing, data analysis and verification.

**Additional file 2: Figure S1.** Effect of *T. gondii* infection on total sperm count.

**Additional file 3: Figure S2.** Identification of infection in experimental mice.

## Data Availability

We have uploaded the transcriptome data to the Sequence Read Archive (SRA) database, submission: SUB5863608, accession ID: PRJNA552423

## Abbreviations

PRU: Prugniaud
DEGs: differentially expressed genes
FDR: false discovery rate
FC: fold change
GO: Gene Ontology
AsAb: anti-sperm antibodies
RNA-seq: RNA sequencing
Gb: gigabases
FPKM: fragment per kilobase of exon model per million mapped reads
SAM: sequence alignment/map format
GTF: gene transfer format
UCSC: University of California Santa Cruz
FRP: horseradish peroxidase
DAB: diaminobenzidine
ECL: enhanced chemiluminescence
KEGG: Kyoto Encyclopedia of Genes and Genomes
MGI: mouse genome informatics
RIN: RNA integrity number
PV: parasitophorous vacuoles
