# Supplementary material for: Transcriptomic analysis of reproductive damage in the epididymis of male Kunming mice induced by chronic infection of Toxoplasma gondii PRU strain
Source: Parasit Vectors. 2019 Nov 8;12:529. doi: 10.1186/s13071-019-3783-2 (PMC6839085; doi:10.1186/s13071-019-3783-2)
Supplement: Supplementary file 1 — Additional file 1: Text S1. Detailed methods of transcriptome sequencing, data analysis and verification. [file 13071_2019_3783_MOESM1_ESM.docx]

**Additional file 1: Text S1. Methods**

**Study population and experiment set-up**

Thirty specific pathogen free eight weeks Kunming male mice were purchased from the Laboratory Animal Center of Guangdong Province. 15 mice were subjected as the experiment groups, and 15 mice were subjected as the control group (to reduce individual differences, we set the epididymal tissue of 5 mice as a biological replicate, the experiment group and the control group were repeated three times). Mice in the experimental group were inoculated with four cysts of *T. gondii* PRU strain diluted with normal saline to 0.5 mL through intragastric administration route. Meanwhile, the control groups were given the same amount of normal saline only. The male mice were eliminated at 35 days post infection. Under sterile condition, the epididymides were harvested. Under the microscope, the peripheral adipose tissue and blood vessels of the harvested epididymides were carefully removed. The processed epididymides were subjected to quick freezing by storing them in liquid nitrogen at -80℃ for subsequent analysis.

**cDNA library construction and sequencing**

Total RNA was extracted using Trizol reagent (Invitrogen, CA, USA) following the manufacturer’s procedure. The total RNA quantity and purity were estimated by Bioanalyzer 2100, using RNA 6000 Nano LabChip Kit (Agilent, CA, USA) with RIN number >7.0. Approximately, 10 ug of total RNA representing a specific adipose type was subjected to isolate Poly (A) mRNA with poly-T oligo-attached magnetic beads (Invitrogen, CA, USA). Following purification, mRNA was fragmented into small pieces using divalent cations under elevated temperature. Then, the cleaved mRNA fragments were reverse-transcribed to create the final cDNA library by the protocol for the mRNA-Seq sample preparation kit (Illumina, San Diego, USA); the average insert size for the paired-end libraries was 300bp (±50bp). Afterwards, we performed the paired-end sequencing on an Illumina Hiseq 2000/2500 (LC Sciences, USA) following the vendor’s recommended protocol.

**Sequences primary analysis and database submission**

A cDNA library constructed by Oligo-capping technology from the pooled RNA from the epididymis samples of the mouse was sequenced. Using the Illumina paired-end RNA-seq approach, 33 million paired-end reads were produced. Initially, the sequencing yielded 48.17 Gigabases (Gb) before assembly. After removing and trimming the low-quality reads (1, reads containing sequencing adaptors; 2, reads containing sequencing primer; 3, nucleotide with q quality score lower than 20), a total of 47.21 Gb of cleaned, paired-end reads were produced. The raw sequence data were submitted to the NCBI.

**RNA-Seq reads mapping**

Reads of sample A and sample B to the UCSC (<http://genome.ucsc.edu/>) mouse reference genome were aligned using TopHat package, which initially removes a portion of the reads based on quality information accompanying each read and then maps the reads to the reference genome. TopHat allows multiple alignments per reading (up to 20 by default) and a maximum of two mismatches when mapping the reads to the reference. TopHat builds a database of potential splice junctions and confirms these by comparing the previously unmapped reads against the database of putative junctions.

**Transcript abundance estimation and differentially expressed testing**

The aligned read files were processed by Cufflinks, which uses the normalized RNA-seq fragment counts to measure the relative abundances of the transcripts. The unit of measurement is fragment per Kilobase of exon model per Million mapped reads (FPKM). The reference GTF annotation file used in Cufflinks was downloaded from the UCSC database. Cufflink was used to de novo assemble the transcriptome at first. Secondly, the Cuffmerge was used to merge all transcripts of sample A and B to generate unique transcripts. The downloaded UCSC GTF file was passed to Cuffdiff along with the original alignment (SAM) files produced by TopHat. Cuffdiff re-estimates the abundance of the transcripts listed in the GTF file using alignments from the SAM file, and concurrently test for a different expression. Only the comparisons with “q value” less than 0.01 and status marked as “OK” in the Cuffdiff output were regarded as showing differential expression.

**Validate the expression level of mRNA by qPCR analysis**

A Real-time Quantitative PCR Detecting System (qPCR) analysis was used to verify gene expression from the RNA-seq analysis. The total RNA was extracted from the epididymis of chronic infection using the RNAiso Plus (Takara, Dalian, China) following the manufacturer’s protocols. The final total RNA obtained was resuspended in RNase-free water. The concentration and purity of the total RNA were measured using an ultra-microspectrophotometer (Thermo, Scientific Nanodrop 2000, Waltham, MA, USA). cDNA was synthesized using the SYBR PrimeScript™ RT Master Mix (Perfect Real Time) Kit (TaKaRa, Dalian, China). Gene-specific qPCR primers were designed based on reference UniGenes sequences with Premier 5.0 software (Premier Biosoft International, Palo Alto, CA, USA), gene-specific primers for qPCR and gene annotation were listed in Table 6. qPCR was performed on a Rotor-Gene Q (Qiagen) real-time system using SYBR Green master mix (SYBR Premix Ex Tag TMII; TaKaRa Bio; http://www.TaKaRa-bio.com) according to the manufacturer’s instructions (TaKaRa, Biotechnology). The thermal profile for the qPCR was 5 min at 95 ℃ followed by 40 cycles of 30 s at 95 ℃ and 1 min at 60 ℃. Each sample was tested in triplicates. Relative gene expression was calculated using the formula 2^–△△Ct^. For normalization of gene expression, *β-actin* was used as an internal standard of mRNA expression. The blank control was used as a reference sample, which was set to 1.

**Western blotting**

Genes of interest were selected for further verification by using western blotting, *Piwil2* (piwi-like RNA-mediated gene silencing 2)*, Spata18* (spermatogenesis associated 18) *and Tnfsf10* (tumor necrosis factor superfamily, member 10) three DEGs from eight genes, which identified by qPCR assay. Similarly, using β-actin as a reference. The pre-processed protein sample was separated on a 10% SDS-PAGE at 120 V and transferred to a Nylon membrane (Roche, Indianapolis, USA) for 45 min at 150 mA. The membrane was blocked overnight at 4℃ in 0.1% Tween 20-PBS containing 5% non-fat dried milk and incubated with *β*-actin mouse monoclonal antibodies (NEOBIOSCIENCE, China. 1:500 dilution), specific mouse anti-Piwil2 (1:800, Abcam), rabbit anti- Spata18 (1:1000, Abcam) and mouse anti- Tnfsf10 (1:400, Abcam) antibodies. The membranes were washed three times and probed with goat anti-mouse or goat anti-rabbit IgG conjugated to horseradish peroxidase (HRP) (Tiangen Biotech, Beijing Co. Ltd., China) at 1:2500 dilution. The membrane was visualized with diaminobenzidine (DAB) substrate solution (Tiangen Biotech, Beijing Co. Ltd., China). The image was analyzed by the ECL plus Western blotting detection system (Tiannon, Shanghai, China).

**Data analysis**

To ensure the authenticity of up-regulation or down-regulation gene, we used P-value < 0.05, |log2 (fold change)|≧1 and FDR < 0.05 as screening conditions. To understand the biological functions of significantly altered genes, DEGs were analyzed by GO bioinformatics tool including biological process, molecular function, and cellular component. [Kyoto Encyclopedia of Genes and Genomes](http://www.so.com/link?url=http%3A%2F%2Fwww.kegg.jp%2F&q=KEGG%E5%85%A8%E7%A8%8B&ts=1494938157&t=bd8dc5f7aef9f3a4ae6e16852da756b&src=haosou) (KEGG) Database (http://www.genome.jp/kegg/) was used providing information on biological pathway analysis, and the gene sequence, while functional information was obtained by using MGI (http://www.informatics.jax.org/) and Ensembl (http://www.ensembl.org/index.html) Databases. Data were reported as a mean ± standard deviation. The null hypotheses that mean identity parameters compared here values were not different between groups were tested by using the Student’s t-test. Statistical analysis was carried out using statistical software SPSS 18.0. Values of *p* < 0.05 were considered significant.
